# Phenotypic and haplotypic profiles of insecticide resistance in populations of *Aedes aegypti* larvae (Diptera: Culicidae) from central Lao PDR

**DOI:** 10.1186/s41182-021-00321-3

**Published:** 2021-04-21

**Authors:** Takaki Shimono, Seiji Kanda, Pheophet Lamaningao, Yuki Murakami, Andrew Waleluma Darcy, Nobuyuki Mishima, Somchit Inthavongsack, Odai Soprasert, Thonelakhanh Xaypangna, Toshimasa Nishiyama

**Affiliations:** 1 Department of Hygiene and Public Health, Kansai Medical University, Hirakata, Osaka, Japan; 2 Regenerative Research Center for Intractable Diseases, Kansai Medical University, Hirakata, Osaka, Japan; 3 Station of Malariology, Parasitology, and Entomology, Khammouane Provincial Health Department, Thakhek, Khammouane Province Lao PDR; 4 Khammouane Provincial Health Department, Thakhek, Khammouane Province Lao PDR

**Keywords:** *Aedes aegypti*, Dengue, Insecticide resistance, Bioassay, Synergist, *kdr* mutation, P450s, Lao PDR

## Abstract

**Background:**

*Aedes aegypti*, which is widely distributed in the Lao People’s Democratic Republic (PDR), is the primary vector of arboviral diseases. Chemical insecticides have been intensively used to eliminate mosquito-borne diseases, resulting in the development of insecticide resistance. However, little is known about the insecticide resistance of mosquito populations in Lao PDR and the mechanisms responsible for it, which have important implications for vector management programs. Here, we examined the phenotypic and haplotypic profiles of insecticide resistance in populations of *Ae. aegypti* larvae from central Lao PDR.

**Methods:**

*Ae. aegypti* larvae were collected from four sites in Lao PDR, and their susceptibility to temephos, deltamethrin, permethrin, and *Bacillus thuringiensis israelensis* (*Bti*) was tested using larval bioassays. Synergistic tests were also conducted to evaluate the activity of insecticide-metabolizing enzymes in the larvae. Deltamethrin-resistant and Deltamethrin-susceptible larvae were then genotyped for knockdown resistance (*kdr*) mutations to determine the associations between each genotype and resistance.

**Results:**

*Ae. aegypti* larvae from central Lao PDR were considered to be “resistant” (<98% mortality) to organophosphates and pyrethroids. The bio-insecticide *Bti* remains effective against such larvae. The resistance mechanisms of *Ae. aegypti* larvae were found to vary among populations, especially for pyrethroid resistance. *Kdr* mutations were significantly associated with deltamethrin resistance in *Ae. aegypti* from the Xaythany population. In contrast, synergist assays with piperonyl butoxide suggested that cytochrome P450 monooxygenases played an important role in the resistance seen in the Khounkham and Thakhek populations.

**Conclusion:**

This study obtained information that will aid the design and implementation of insecticide-based vector management of *Ae. aegypti* in central Lao PDR. *Ae. aegypti* larvae from central Lao PDR were highly susceptible to *Bti*, while they were resistant to temephos at a diagnostic dose of 0.0286 mg/L. Given the limited number of insecticides that are approved for vector control, it is important to alternate between temephos and other larvicides, such as *Bti* and pyriproxyfen. The differences in pyrethroid resistance mechanisms seen among the *Ae. aegypti* populations highlight the need to tailor vector-control strategies to each region to increase the success of dengue control in Lao PDR.

**Supplementary Information:**

The online version contains supplementary material available at 10.1186/s41182-021-00321-3.

## Background

*Aedes aegypti* (Linnaeus), an aggressive and daytime-biting mosquito, is a species of significant medical and public health importance, due to its ability to transmit a multitude of arboviruses. It played an important role in recent dengue outbreaks worldwide. It is highly adapted to rural, semi-urban, and urban environments, where it can breed in a variety of artificial containers, and females feed on human blood. About 2.5 billion people in more than 100 tropical and subtropical countries where *Ae. aegypti* is found are at risk of contracting dengue [1]. Of this at-risk population, 1.8 billion live in countries in Asia or the Pacific region. Since no effective vaccines or therapeutics for arboviral diseases are available, vector-control measures targeting *Ae. aegypti* have been used to minimize the incidence rates of such infections [2, 3].

Attempts to control *Ae. aegypti* have been based on reducing the number of larvae by eliminating larval habitats, e.g., by destroying or disposing of unused water-holding containers, such as jars, buckets, and old tires, and/or emptying them. Besides the mechanical elimination of mosquito habitats, the application of larvicidal treatments to larval habitats and outdoor and indoor adulticide-based spatial spraying operations carried out by house owners and/or local authorities have also been employed for many decades worldwide [2]. For both larvicidal and adulticidal purposes, organophosphates, such as malathion and temephos, and pyrethroids, such as deltamethrin, etofenprox, and permethrin, are commonly used [4]. However, the massive and widespread use of chemical insecticides has resulted in the development of insecticide resistance in dengue vectors, particularly *Ae. aegypti*, in many countries, which represents an operational challenge for vector-control programs [2, 5].

*Ae. aegypti* has evolved and developed various insecticide-resistance mechanisms, which mainly involve reductions in target-site sensitivity and enhanced metabolic detoxification. Metabolic detoxification is associated with upregulated expression of three major families of enzyme genes, i.e., the genes for cytochrome P450 monooxygenases (P450s), carboxylesterases (COEs), and glutathione S-transferases (GSTs). Another key resistance mechanism is target-site insensitivity caused by non-synonymous mutations in the gene for the voltage-gated sodium channel targeted by organochlorine and pyrethroid insecticides, which can arise through knockdown resistance (*kdr*) [5–10]. Several *kdr* mutations, such as V1016G and F1534C, have been identified in *Ae. aegypti*, and associations between these mutations and pyrethroid resistance have been confirmed [11–13].

Lao People’s Democratic Republic (Lao PDR) is one of the countries in Southeast Asia affected by dengue, and all four dengue virus serotypes circulate in the country on a 2–5-year cyclical pattern [14–16]. The primary dengue vector, *Ae. aegypti*, is widely distributed throughout Lao PDR [17]. Compared with neighboring countries, there is a lack of studies on insecticide resistance in *Ae. aegypti* in Lao PDR, which could aid the establishment of effective and locally adapted vector-control systems.

In this study, we conducted bioassays, synergist assays, and genotyping of *kdr* alleles to investigate the status of insecticide resistance and the associated molecular mechanisms in four field populations of *Ae. aegypti* larvae from suburban or rural areas along the Mekong River or its tributaries in central Lao PDR.

## Methods

## Sampling

**Fig. 1:**
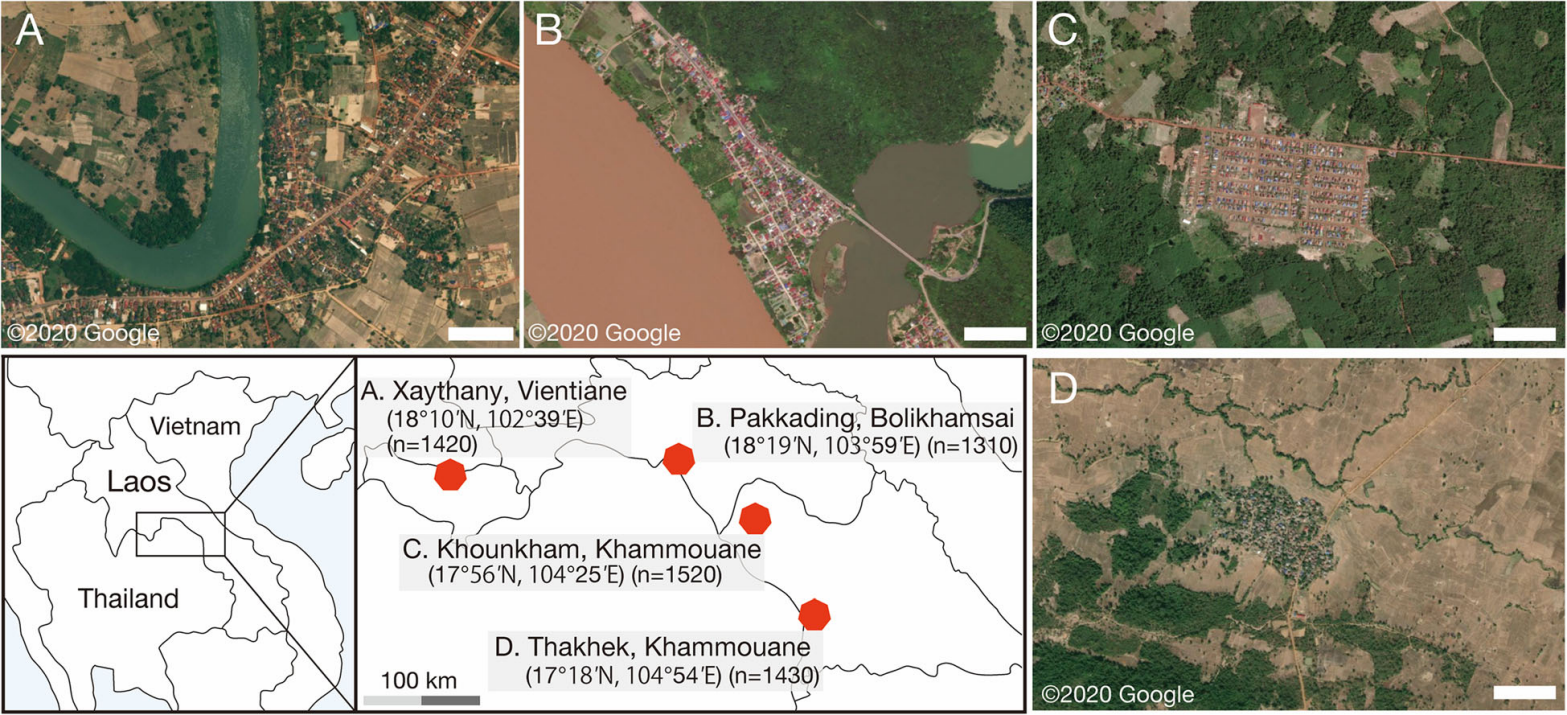
Sampling sites and status of mosquito larvae at each site in central Lao PDR. **a**, **b** Suburban townships along the national highway in Xaythany, Vientiane, and Pakkading, Bolikhamsai. **c** A rural village surrounded by a forest in Khounkham, Khammouane. **d** A rural village surrounded by rice fields and a forest in Thakhek, Khammouane; n: number of *Aedes* spp. larvae collected from each population; scale bars A–D 250 m

The *Aedes* spp. larvae used in this study were collected between January and October 2019 in rural villages or suburban townships in four different locations in Bolikhamsai Province, Khammouane Province, or the capital Vientiane (Fig. 1). The larvae were collected from water storage containers, such as cement tanks and jars. Verbal permission was obtained from each householder to conduct entomological collections on their premises.

### Larval bioassay

The following analytical-standard or technical-grade insecticides were used for the larval bioassays: deltamethrin (> 97.0%; Tokyo Chemical Industry Co., Ltd., Tokyo, Japan), permethrin (≥ 90.0%; Sigma-Aldrich, Inc., MO, USA), temephos (≥ 95.0%; Sigma-Aldrich, Inc.), and *Bacillus thuringiensis israelensis* (*Bti*) (VectoBac WG, 3000 international toxic units/mg, strain H-14) (Sumitomo Chemical Co., Ltd., Tokyo, Japan). The stock solutions of pyrethroids and temephos were prepared in dimethyl sulfoxide (DMSO) (Fujifilm Corp., Tokyo, Japan). The DMSO concentration of each final solution was < 0.1%, which did not affect the survival rate of *Aedes* spp. in the larval bioassays (data not shown). The stock solution of *Bti* was prepared in distilled water.

The diagnostic dose of an insecticide is a dose that is two-fold higher than the lethal concentration and kills 99% of an insecticide-susceptible reference strain, such as Bora-Bora or the US Department of Agriculture strain, in World Health Organization (WHO) larval bioassays. However, the diagnostic doses of most of the insecticides used against *Ae. aegypti* have not yet been determined. Furthermore, unfortunately no reference strains were available for this study, and hence, the diagnostic doses of the examined insecticides could not be established. Therefore, the larval bioassays performed in the present study involved diagnostic doses of deltamethrin (0.003 mg/L), permethrin (0.008 mg/L), temephos (0.0286 mg/L), and *Bti* (1.1406 mg/L) that were established using the Bora-Bora strain in previous studies [18, 19].

Bioassays were carried out using wild mosquito larvae obtained from the sampling site on the day of collection. Each bioassay was conducted using a disposable, transparent, 180-mL plastic cup, containing 99 mL of distilled water, and 1 mL of insecticide solution at the desired concentration. Twenty-five to 30 third- to fourth-instar larvae were randomly selected and transferred to the cup for 10–15 min before the addition of the insecticide solution. Three to 6 replications were carried out for each insecticide for all four field strains. Mortality was assessed after 24 h of exposure at room temperature. Larvae that sank to the bottom of the cup and could not swim or float, or were paralyzed were judged to be knocked-down larvae.

To evaluate the effectiveness of insecticide detoxification by the mosquitoes, three synergists, ethacrynic acid (EA, ≥ 95.0%; Sigma-Aldrich, Inc.), piperonyl butoxide (PBO, 98.0%; Fujifilm Corp.), and triphenyl phosphate (TPP, > 99.0%; Tokyo Chemical Industry Co., Ltd.), were used to conduct synergist tests. Sublethal doses of EA (0.1 mg/L), PBO (5 mg/L), and TPP (1 mg/L), which were determined in previous studies, were used in these tests [18, 19]. The larval synergist tests were conducted similarly to the larval bioassays, although they involved an additional step, in which the synergist and insecticide was mixed at a 1:1 ratio before the larvae were exposed to them. Each of the synergists was combined with each of the insecticides, except *Bti*.

To investigate the associations between deltamethrin resistance and *kdr* mutations, after the bioassays each larva was examined under a dissecting microscope to identify *Ae. aegypti*, according to the characteristics described in a previous study [20], and then was stored in a 1.5-mL plastic tube containing ethanol solution until it was used for the subsequent genetic analysis.

### Knockdown resistance (*kdr*) genotyping

Direct DNA sequencing was conducted to detect the L982W, S989P, I1011M/V, V1016G, T1520I, and F1534C point mutations in *Ae. aegypti*. Each population contained a maximum of 25 live and 25 dead larvae, which were randomly selected from among those used for the deltamethrin bioassay, and the total number of extracts used for the *kdr* genotyping was 163.

Genomic DNA was extracted from individual mosquitoes using Isogenome (Nippon Gene Co., Ltd., Tokyo, Japan), according to the manufacturer’s instructions, in a final volume of 30 μL of distilled water. The polymerase chain reaction (PCR) was performed using TaKaRa Ex Taq, hot start version (Takara Bio, Shiga, Japan), under the following conditions: initial denaturation was conducted at 94 **°**C for 2 min; followed by 5 cycles of 98 **°**C for 10 s, 60 **°**C for 30 s, and 72 **°**C for 1 min; 30 cycles of 98 **°**C for 10 s, 58 **°**C for 30 s, and 72 **°**C for 1 min; and a final extension step of 72 **°**C for 5 min. The PCR products were purified as described above, and then the DNA sequencing was carried out using the BigDye Terminator, version 3.1, cycle sequencing kit (ThermoFisher Scientific, Inc., MA, USA), according to the manufacturer’s instructions. The amplification and partial sequencing of the target regions were conducted using the primers shown in Table 1. Direct DNA sequencing was performed using the 3130 × l analyzer (ThermoFisher Scientific, Inc.). The electropherograms of the replacement amino acids found in the target regions were analyzed with the MEGA 7.0 software [22].

**Table 1:** The primers used for the *kdr* genotyping

Amplified region	Primer name	Primer sequence (5′–3′)
L982W, S989P, I1011M/V, and V1016G	AaS CF1 (PCR)	AGACAATGTGGATCGCTTCC
	AaS CR4 (PCR and DNA sequencing)	GGACGCAATCTGGCTTGTTA
	AaS CF3 (DNA sequencing)	GTGGAACTTCACCGACTTCA
T1520I and F1534C	AaS CF7 (PCR and DNA sequencing)	GAGAACTCGCCGATG AACTT
	AaS CR7 (DNA sequencing)	GACGACGAAATCGAACAGGT
	AaS CR8 (PCR)	TAGCTTTCAGCGGCTT CTTC

The primer sequences were obtained from a study by Kawada et al. 2016 [21]

### Phenotype-haplotype associations

To assess the roles of the S989P, V1016G, T1520I, and F1534C mutations in deltamethrin resistance, we conducted a phenotype-haplotype analysis by comparing the distributions of these *kdr* mutations between dead and alive mosquito larvae that had been exposed to deltamethrin.

### Statistical analyses

Statistical analyses were performed using GraphPad Prism, version 8.10 (San Diego, CA, USA, www.graphpad.com), and EZR, which is based on R and R commander [23]. Fisher’s exact test with Holm’s correction for multiple comparisons was used to compare the resistant allele frequency of S989P, V1016G, and F1534C among the *Ae. aegypti* larvae in the four field populations. One-way analysis of variance (ANOVA) followed by Tukey’s multiple comparisons test was used to compare the larval mortality rates produced by temephos, deltamethrin, and permethrin in each population. One-way ANOVA followed by Dunnett’s multiple comparisons test was used to compare larval mortality rates between the groups treated with insecticides and those treated with combinations of insecticides and synergists. Odds ratios (ORs) and 95% confidence intervals (95% CI) were estimated using Fisher’s exact test to assess the associations between *kdr* mutations and resistant phenotypes.

## Results

### Bioassays

A total of 5680 *Aedes* spp. larvae were collected from four populations (Fig. 1). In the bioassays, we identified *Aedes* spp. larvae based on their morphological features. *Ae. aegypti* was the predominant larval species in the Xaythany, Khounkham, and Thakhek populations. In contrast, *Ae. albopictus* accounted for a large proportion of the Pakkading population (data not shown). Accordingly, the samples collected from Pakkading were excluded from the statistical analyses of the larval bioassays due to the insufficient *Ae. aegypti* sample size.

**Fig. 2:**
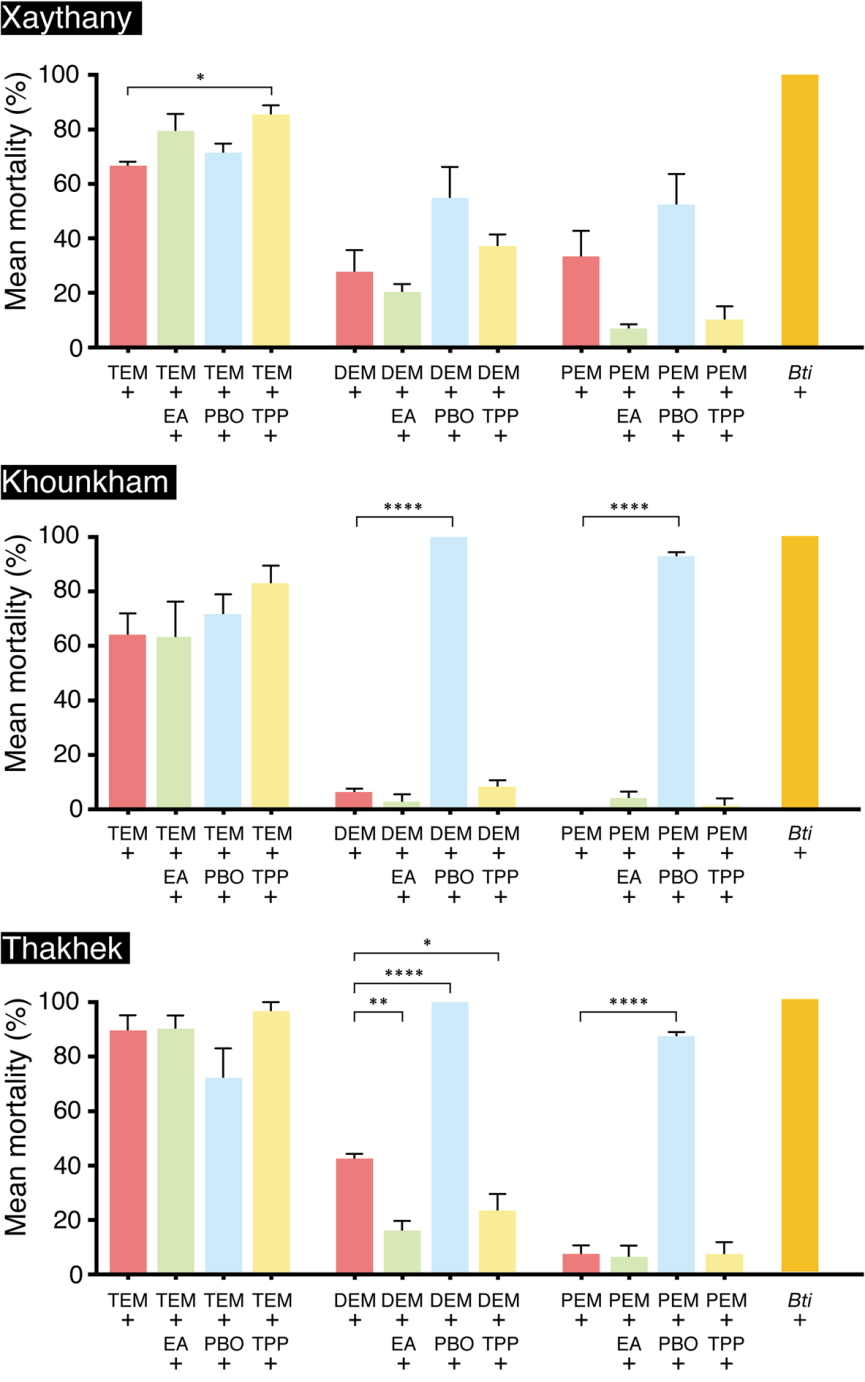
Comparison of the mortality rates of *Ae. aegypti* larvae treated with different insecticides and synergists. Ordinary one-way ANOVA and Dunnett’s multiple comparisons test, with a single pooled variance, were used for the analyses. Significant differences in mortality rates between the groups treated with an insecticide alone and those treated with an insecticide plus a synergist is indicated by asterisks. *p < 0.05, **p < 0.01, ****p < 0.0001; TEM: temephos, DEM: deltamethrin, PEM: permethrin

The mortality rates of the *Ae. aegypti* larvae treated with temephos, deltamethrin, or permethrin in the three populations were < 90% at the diagnostic doses employed in the bioassay. According to the WHO standards, an *Ae. aegypti* population is classified as “resistant” if its morality rate is < 90%, as “suspected” if its mortality rate ranges from 90% to 98%, and as “susceptible” if its mortality rate is > 98% [24]. The results of the bioassays indicated that the *Ae. aegypti* from the three sites were “insecticide-resistant.” On the other hand, the bio-insecticide *Bti* resulted in 100% mortality in all populations (Fig. 2, Table S1).

When the larvae from the three sites were treated with temephos, their mortality rates ranged from 64.0 to 89.7%. In contrast, the mortality rates produced by deltamethrin and permethrin were significantly lower than those produced by temephos in all of the *Ae. aegypti* populations (Fig. 2, Table S1).

Significant differences in the temephos- and pyrethroid-related mortality rates were observed among the *Ae. aegypti* populations. The mortality rates produced by temephos and deltamethrin in the *Ae. aegypti* population in Thakhek were significantly higher than those seen in the *Ae. aegypti* population in Khounkham (*p* < 0.05 and *p* < 0.01, respectively). On the other hand, the *Ae. aegypti* mortality rate produced by permethrin was higher in Xaythany than in Khounkham and Thakhek (*p* < 0.01 and *p* < 0.05, respectively) (Table S1).

### Synergist tests

Synergist tests were performed to investigate the role of insecticide detoxification in insecticide resistance in *Ae. aegypti*. The treatment of *Ae. aegypti* larvae with any of the three synergists alone did not affect the survival rate of the larvae (Table S1). The synergist tests showed that the mortality rate produced by temephos plus TPP was significantly higher than that produced by temephos alone in the *Ae. aegypti* population from Xaythany, while none of the other three synergists (EA, TPP, or PBO) increased the temephos-induced mortality rate of the *Ae. aegypti* larvae from any of the three locations (Fig. 2, Table S1).

PBO treatment led to marked increases in the *Ae. aegypti* mortality rates produced by deltamethrin and permethrin in Khounkham and Thakhek. On the other hand, the mortality rates produced by these insecticides were slightly, but not significantly, increased by PBO in Xaythany. When EA or TPP was used in conjunction with deltamethrin or permethrin, no significant changes in the mortality rate were found in any of the *Ae. aegypti* populations (Fig. 2, Table S1).

### Knockdown resistance (*kdr*) genotyping

Genotyping of the sodium channel gene of *Ae. aegypti* to detect the L982W, S989P, I1011M/V, V1016G, T1520I, and F1534C mutations was carried out on individuals (163 in total), which were collected from four different locations, including Pakkading, and subjected to a bioassay.

No L982W or I1011M/V amino acid mutations were observed in any of the assayed individuals, while S989P, V1016G, T1520I, and F1534C amino acid mutations were found in some individuals (accession numbers LC605641 to LC605645).

**Fig. 3:**
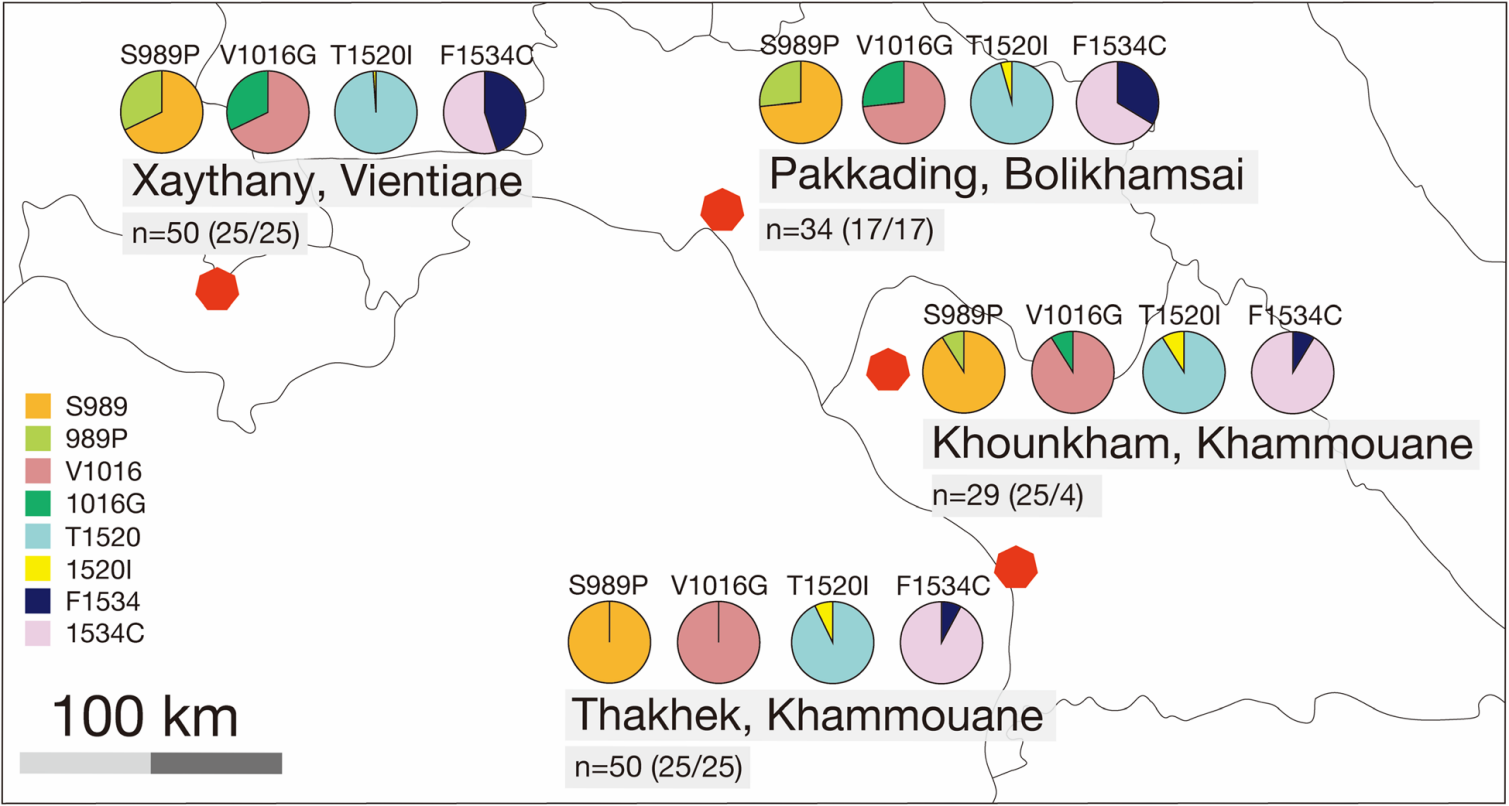
Distribution of *kdr* alleles among field populations of *Ae. aegypti* that were subjected to bioassays. In the Khounkham population, since only four mosquitoes that were susceptible to deltamethrin were available for the bioassay, only four susceptible mosquitoes were subjected to *kdr* genotyping. n: number of samples analyzed (alive/dead)

Focusing on the presence and frequency of the S989P, V1016G, T1520I, and F1534C mutations in the *Ae. aegypti* at each site, the F1534C mutation was found in all of the examined populations. The allelic frequency of 1534C was relatively high, ranging from 55.0 to 92.0%. It was particularly high in the populations from Khounkham (91.4%) and Thakhek (92.0%). The T1520I mutation was detected in all of the examined populations, and the allelic frequency of this mutation ranged from 1.0% to 8.6%. The S989P and V1016G mutations were always found together. The allelic frequencies of 989P and 1016G were 32.0% and 31.0%, respectively, in the population from Xaythany, and 26.5% in the population from Pakkading, while it was 8.6%; i.e., a quarter of that seen in the population from Xaythany, in the population from Khounkham, and no such mutations were observed in the population from Thakhek (Fig. 3).

Significant differences in the frequencies of the 989P and 1016G alleles were found among the populations, except between the Xaythany and Pakkading populations (*p* < 0.05), and significant differences in the frequency of the 1534C allele were found among the populations, except between the Xaythany and Pakkading populations, and the Khounkham and Thakhek populations (*p* < 0.05). No significant differences in the frequency of the 1520I allele were found among the populations (*p* > 0.05) (Table S2).

We identified thirteen genotypes and five haplotypes in the current study (Table 2, Table S3). In addition to detecting a single *kdr* mutation, namely F1534C (S-V-T-C haplotype), three co-occurring *kdr* mutations, V1016G + S989P (P-G-T-F haplotype), T1520I+F1534C (S-V-I-C haplotype), and V1016G + F1534C+S989P (P-G-T-C haplotype), were also found. The frequencies of these multiple-mutant haplotypes (P-G-T-F, S-V-I-C, and P-G-T-C) in the *Ae. aegypti* larvae from the four populations ranged from 0 to 20.0%, 2.0 to 13.8%, and 0 to 29.4%, respectively. No single S989P, V1016G, or T1520I *kdr* mutations were detected.

**Table 2:** Frequency of thirteen *kdr* genotypes in *Ae. aegypti* larvae collected from four populations

Haplotype	Genotype	Xaythany			Pakkading			Khounkham			Thakhek		
		No. of samples analyzed			No. of samples analyzed			No. of samples analyzed			No. of samples analyzed		
		Alive	Dead	Total	Alive	Dead	Total	Alive	Dead	Total	Alive	Dead	Total
		25	25	50	17	17	34	25	4	29	25	25	50
S-V-T-F	S/S + V/V + T/T + F/F	0.00 (0/25)	0.04 (1/25)	0.02 (1/50)	0.00 (0/17)	0.12 (2/17)	0.06 (2/34)	0.00 (0/25)	0.00 (0/4)	0.00 (0/29)	0.04 (1/25)	0.08 (2/25)	0.06 (3/50)
S-V-T-C	S/S + V/V + T/T + F/C	0.20 (5/25)	0.44 (11/25)	0.32 (16/50)	0.12 (2/17)	0.24 (4/17)	0.18 (6/34)	0.08 (2/25)	0.00 (0/4)	0.07 (2/29)	0.00 (0/25)	0.12 (3/25)	0.06 (3/50)
	S/S + V/V + T/T + C/C	0.16 (4/25)	0.40 (10/25)	0.28 (14/50)	0.35 (6/17)	0.29 (5/17)	0.32 (11/34)	0.64 (16/25)	0.75 (3/4)	0.66 (19/29)	0.92 (23/25)	0.64 (16/25)	0.78 (39/50)
S-V-I-C	S/S + V/V + T/I + F/C	0.00 (0/25)	0.00 (0/25)	0.00 (0/50)	0.00 (0/17)	0.06 (1/17)	0.03 (1/34)	0.00 (0/25)	0.00 (0/4)	0.00 (0/29)	0.00 (0/25)	0.00 (0/25)	0.00 (0/50)
	S/S + V/V + T/I + C/C	0.00 (0/25)	0.04 (1/25)	0.02 (1/50)	0.00 (0/17)	0.12 (2/17)	0.06 (2/34)	0.12 (3/25)	0.25 (1/4)	0.14 (4/29)	0.04 (1/25)	0.08 (2/25)	0.06 (3/50)
	S/S + V/V + I/I + C/C	0.00 (0/25)	0.00 (0/25)	0.00 (0/50)	0.00 (0/17)	0.00 (0/17)	0.00 (0/17)	0.00 (0/25)	0.00 (0/4)	0.00 (0/29)	0.00 (0/25)	0.08 (2/25)	0.04 (2/50)
P-G-T-F	P/P + V/G + T/T + F/F	0.04 (1/25)	0.00 (0/25)	0.02 (1/50)	0.00 (0/17)	0.00 (0/17)	0.00 (0/17)	0.00 (0/25)	0.00 (0/4)	0.00 (0/29)	0.00 (0/25)	0.00 (0/25)	0.00 (0/50)
	P/P + G/G + T/T + F/F	0.32 (8/25)	0.04 (1/25)	0.18 (9/50)	0.06 (1/17)	0.06 (1/17)	0.06 (2/34)	0.00 (0/25)	0.00 (0/4)	0.00 (0/29)	0.00 (0/25)	0.00 (0/25)	0.00 (0/50)
P-G-T-C	S/P + V/G + T/T + F/C	0.12 (3/25)	0.00 (0/25)	0.06 (3/50)	0.24 (4/17)	0.06 (1/17)	0.15 (5/34)	0.12 (3/25)	0.00 (0/4)	0.10 (3/29)	0.00 (0/25)	0.00 (0/25)	0.00 (0/50)
	S/P + G/G + T/T + F/C	0.00 (0/25)	0.04 (1/25)	0.02 (1/50)	0.06 (1/17)	0.00 (0/17)	0.03 (1/34)	0.00 (0/25)	0.00 (0/4)	0.00 (0/29)	0.00 (0/25)	0.00 (0/25)	0.00 (0/50)
	P/P + V/G + T/T + C/C	0.04 (1/25)	0.00 (0/25)	0.02 (1/50)	0.06 (1/17)	0.00 (0/17)	0.03 (1/34)	0.00 (0/25)	0.00 (0/4)	0.00 (0/29)	0.00 (0/25)	0.00 (0/25)	0.00 (0/50)
	P/P + G/G + T/T + F/C	0.12 (3/25)	0.00 (0/25)	0.06 (3/50)	0.06 (1/17)	0.06 (1/17)	0.06 (2/34)	0.00 (0/25)	0.00 (0/4)	0.00 (0/29)	0.00 (0/25)	0.00 (0/25)	0.00 (0/50)
	P/P + G/G + T/T +C/C	0.00 (0/25)	0.00 (0/25)	0.00 (0/50)	0.06 (1/17)	0.00 (0/17)	0.03 (1/34)	0.04 (1/25)	0.00 (0/4)	0.03 (1/29)	0.00 (0/25)	0.00 (0/25)	0.00 (0/50)

The sites of the *kdr* mutations are underlined

### Associations between *kdr* mutations and resistant phenotypes

The genotyping of the *kdr* mutations was conducted on a group of living individuals and a group of dead individuals (a maximum of 25 individuals in each group) for each site. These individuals were subjected to a bioassay to verify whether a relationship existed between resistance to deltamethrin and particular results revealed that the S-V-T-C haplotype (a single 1534C mutant) was not related to resistance to deltamethrin in any of the populations or in the whole study population (OR < 1.0, *p* > 0.05; Table 3). On the other hand, the P-G-T-F haplotype (V1016G + S989P) was significantly associated with resistance to deltamethrin in the population from Xaythany and in the whole study population (OR > 1.0, *p* < 0.01) (Table 4). In addition to the P-G-T-F haplotype, the P-G-T-C haplotype (V1016G + F1534C+S989P) exhibited OR for resistance to deltamethrin of 9.33 (*p* = 0.049) in the population from Xaythany and 5.90 (*p* = 0.002) in the whole study population, which confirmed that these haplotypes are significantly related to resistance to deltamethrin (Table 5).

**Table 3:** Associations between the S-V-T-C haplotype and deltamethrin resistance among *Ae. aegypti* larvae collected from four populations

Locality	No. of samples analyzed, alive/dead	Alive			Dead			Odds ratio (95% CI)	Fisher’s exact test (*p* value)
		No. of mosquitoes		Freq. of S-V-T-C	No. of mosquitoes		Freq. of S-V-T-C		
		S-V-T-F and P-G-T-F	S-V-T-C		S-V-T-F and P-G-T-F	S-V-T-C			
Xaythany	18/23	9	9	0.50 (9/18)	2	21	0.91 (21/23)	0.10 (0.2 to 0.56)	0.0046
Pakkading	9/12	1	8	0.89 (8/9)	3	9	0.75 (9/12)	2.67 (0.32 to 38.4)	0.60
Khounkham	18/3	0	18	1.00 (18/18)	0	3	1.00 (3/3)	Not determined	
Thakhek	24/21	1	23	0.96 (23/24)	2	19	0.90 (19/21)	2.42 (0.03 to 3.83)	0.59
Total	69/59	11	58	0.84 (58/69)	7	52	0.88 (52/59)	0.71 (0.27 to 2.04)	0.61

The sites of the *kdr* mutations are underlined

**Table 4:** Associations between the P-G-T-F haplotype and deltamethrin resistance among *Ae. aegypti* larvae collected from four populations

Locality	No. of samples analyzed, alive/dead	Alive			Dead			Odds ratio (95% CI)	Fisher’s exact test (*p* value)
		No. of mosquitoes		Freq. of P-G-T-F	No. of mosquitoes		Freq. of P-G-T-F		
		S-V-T-F, S-V-T-C, and S-V-I-C	P-G-T-F		S-V-T-F, S-V-T-C and S-V-I-C	P-G-T-F			
Xaythany	17/24	9	8	0.47 (8/17)	23	1	0.04 (1/23)	20.4 (2.75 to 234.3)	0.0017
Pakkading	9/15	8	1	0.11 (1/9)	14	1	0.07 (1/15)	1.75 (0.8 to 35.32)	> 0.9999
Khounkham	0/0	0	0	Not determined	0	0	Not determined	Not determined	
Thakhek	0/0	0	0	Not determined	0	0	Not determined	Not determined	
Total	26/39	17	9	0.35 (9/26)	37	2	0.07 (5/71)	9.79 (2.03 to 47.27)	0.0047

The sites of the *kdr* mutations are underlined

**Table 5:** Associations between the P-G-T-C haplotype and deltamethrin resistance among *Ae. aegypti* larvae collected from four populations

Locality	No. of samples analyzed, alive/dead	Alive			Dead			Odds ratio (95% CI)	Fisher’s exact test (*p* value)
		No. of mosquitoes		Freq. of P-G-T-C	No. of mosquitoes		Freq. of P-G-T-C		
		S-V-T-F, S-V-T-C, S-V-I-C and P-G-T-F	P-G-T-C		S-V-T-F, S-V-T-C, S-V-I-C and P-G-T-F	P-G-T-C			
Xaythany	25/25	18	7	0.28 (7/25)	24	1	0.06 (1/25)	9.33 (1.35 to 108.8)	0.049
Pakkading	17/17	9	8	0.47 (8/17)	15	2	0.15 (2/17)	6.67 (1.32 to 34.56)	0.057
Khounkham	24/4	21	4	0.16 (4/25)	4	0	0.00 (0/4)	Not determined	
Thakhek	25/25	25	0	0.00 (0/25)	25	0	0.00 (0/25)	Not determined	
Total	92/71	73	19	0.21 (19/92)	68	3	0.04 (3/71)	5.90 (1.79 to 19.40)	0.002

The sites of the *kdr* mutations are underlined

## Discussion

### Resistance to temephos and *Bti*

In the present study, the diagnostic doses of the insecticides could not be determined using a susceptible reference strain; therefore, the diagnostic doses used in previous studies were adopted. The results of the bioassays carried out under these conditions were largely consistent with those obtained in previous studies of *Ae. aegypti* populations from Lao PDR and other regions of Southeast Asia [17–19]. Specifically, they revealed that all of the *Ae. aegypti* populations from the three sites in central Lao PDR were resistant to temephos (diagnostic dose 0.0286 mg/L), but the insecticide resistance status of the mosquitoes varied among the study sites. Previous studies of populations from Lao PDR and Malaysia have also reported that *Ae. aegypti* is resistant to temephos, but the levels of resistance varied among the study sites [17, 19]. A study conducted in Lao PDR showed that the temephos susceptibility status of *Ae. aegypti* is “moderate”, while the resistance ratios obtained for the central and southern populations at the 50% and 95% lethal concentrations (LC_50_ and LC_95_, respectively), i.e., the RR_50_ and RR_95_ values, were > 3.0, suggesting that these *Ae. aegypti* populations are more resistant to temephos [17]. It should be noted that the temephos formulation recommended by the WHO for controlling mosquito larvae in container habitats contains 1 mg/L of the active ingredient, which is much higher than the diagnostic dose used in this study and the LC_95_ reported in previous studies performed in Lao PDR [2, 17, 25]. Thus, using temephos as recommended in the WHO guidelines might be effective at controlling *Ae. aegypti* larvae in Lao PDR.

All of the *Ae. aegypti* populations from the three sites in central Lao PDR were “susceptible” to *Bti*. In a previous study conducted in Lao PDR, the insecticide susceptibility of *Ae. aegypti* larvae collected from Vientiane to *Bti* was tested. As a result, it was revealed that the RR_50_ and RR_95_ values for each of the populations were < 1.0, suggesting that the larvae were susceptible to *Bti* [26]. This finding supports the results of the current study. The fact that *Bti* has never been used in Lao PDR for vector-control programs and has a different insecticide mode of action to temephos and pyrethroids, which have been used in Lao PDR, is considered to explain the absence of resistance to *Bti* in all of the *Ae. aegypti* populations tested in the current study [26]. In addition, a study involving a field experiment carried out in Malaysia reported that the susceptibility of *Ae. aegypti* larvae to *Bti* did not vary, even though *Bti* had been used intensively for 7 months [27]. Studies performed in Singapore, where *Bti* has been used as a main larvicide for over 10 years, have also reported that larvae collected from the field exhibited no resistance to *Bti* [18, 27]. As mentioned above, the use of *Bti* as a larvicide is considered to be effective in Lao PDR.

Lamaningao et al. [28] conducted a vector-control field trial using SumiLarv®2MR (Sumitomo Chemical Co., Ltd.), which contains pyriproxyfen, an insect growth regulator (IGR) with a different mechanism of action to temephos and *Bti*, between October 2017 and July 2019. Consequently, it was shown that pyriproxyfen-based interventions were effective at controlling the number of *Ae. aegypti* larvae in the studied village. As mentioned above, using temephos as a larvicide according to the WHO standards is expected to have sufficient insecticidal effects in Lao PDR. On the other hand, the continuous use of temephos might strengthen the insecticide resistance of *Ae. aegypti* larvae [18, 27]. Therefore, to allow effective and sustainable temephos usage, it is suggested that the bio-insecticide *Bti* and the IGR pyriproxyfen, which have different mechanisms of action from temephos, and temephos should be used in rotation.

### Pyrethroid resistance

#### Bioassays and synergist testing

The bioassay carried out in the present study demonstrated that the *Ae. aegypti* populations from the three sites exhibited greater resistance to pyrethroids than to temephos. Many studies conducted in Southeast Asia have reported that *Ae. aegypti* is highly resistant to pyrethroids [13, 29–32]. On the other hand, little was known about the resistance of *Ae. aegypti* in Lao PDR to pyrethroids until a 2019 study by Marcombe et al. [17], which suggested that most of the *Ae. aegypti* populations in the northern, central, and southern areas of Lao PDR exhibit resistance to pyrethroids. The current study confirmed that *Ae. aegypti* populations from Lao PDR displayed resistance to pyrethroids.

Furthermore, synergistic tests were conducted to evaluate the activity of insecticide-metabolizing enzymes. As a consequence, it was shown that when PBO was added to pyrethroids the mortality rates of the *Ae. aegypti* populations from Khounkham and Thakhek increased significantly, while no significant change was observed in the mortality rate of the population from Xaythany. When EA and TPP were added to pyrethroids, no significant increase in mortality was observed in any of the *Ae. aegypti* populations. Generally, PBO inhibits the enzyme activity of P450s, while EA and TPP inhibit the enzyme activities of GSTs and COEs [33, 34]. Thus, it is suggested that P450 enzymatic activity might have played a role in the resistance to pyrethroids seen in the Khounkham and Thakhek populations.

In Lao PDR, pyrethroids are commonly used for fogging to control adult mosquitoes rather than as larvicides (Lamaningao, personal communication with Khammouane Provincial Health Department of Lao PDR). It is reported that common mechanism(s) cause insecticide resistance in both larval and adult mosquitoes [35]. Thus, the mechanism responsible for pyrethroid resistance and the resultant larval population phenotypes observed in the current study might have been acquired at the adult stage. However, it should be noted that resistance profiles can also vary between life stages, in contrast to the findings of the study mentioned above [36]. Thus, further adulticide bioassays are required to validate our findings, as was discussed in previous studies [18, 19].

### Knockdown resistance (*kdr*) genotyping

The present study analyzed sodium channel gene mutations, which are related to resistance to pyrethroids in Southeast Asian *Ae. aegypti* populations. As a result, the V1016G mutation was detected at an allele frequency ranging from 0 to 31%. Thus, the frequency of 1016G varied among the populations in the present study. It has been reported that the frequency of this mutant allele was high in *Ae. aegypti* populations from Thailand, Indonesia, and Myanmar, while it was not so high in populations from Vietnam and Lao PDR (0 to 36%) [13, 17, 29–32, 37–39].

Previous studies clarified that the 1534C mutant is widely distributed, and its frequency is high in Southeast Asian *Ae. aegypti* populations [31, 32, 40]. In a previous study, the frequency of the 1534C mutant was found to be high in *Ae. aegypti* populations from various sites in Lao PDR, but not at one southern site, which was consistent with the results of the current study [17].

We identified a new *kdr* mutation, S989P (with V1016G or V1016G + F1534C), for the first time in Lao PDR. The co-occurrence of V1016G + S989P and the V1016G + F1534C + S989P triple mutation have been reported in previous studies conducted in other Southeast Asian countries [11, 13, 31, 41].

### Associations between *kdr* mutations and resistant phenotypes

In the current study, the F1534C mutation was detected at high frequencies in the populations from all four sites; however, no significant association was found between the F1534C mutation and deltamethrin resistance. In the bioassays conducted in a previous study, no association between the F1534C mutation and deltamethrin resistance was observed in *Ae. aegypti* populations from Thailand, which agrees with the results of the present study [32]. Furthermore, neurophysiological experiments, involving *Xenopus* oocyte expression systems, also supported the idea that the F1534C mutation is not related to resistance to type II pyrethroids, such as deltamethrin [12, 42]. However, one limitation of the present study and the study conducted in Thailand [32] is that a single concentration of deltamethrin was used to compare the frequencies of F1534C in bioassays of susceptible and resistant mosquitoes from field populations. In fact, Kushwah et al. [43], who used a similar approach to that adopted in the present study, found that the F1534C mutation was associated with deltamethrin resistance in Indian *Ae. aegypti.* Furthermore, Fan and Scott [44] reported in their study, in which they used a congenic strain of *Ae. aegypti* possessing the F1534C mutation, that this mutation confers 16-fold resistance to deltamethrin. Hence, further investigation is required to evaluate the role of the F1534C mutation in the deltamethrin resistance seen in *Ae. aegypti* in central Lao PDR.

In contrast to the F1534C mutation, the current study suggested that the V1016G mutation in combination with the S989P mutation (the P-G-T-F haplotype) and the S989P+F1534C double mutation (the P-G-T-C haplotype) were linked to deltamethrin resistance in the *Ae. aegypti* population from Xaythany. Both haplotypes result in strong resistance to pyrethroids [11, 12, 42]. Hirata et al. [12] reported that the P-G-T-F haplotype reduced sodium channel sensitivity to permethrin and deltamethrin by 100- and 10-fold, respectively. Furthermore, the P-G-T-C haplotype reduced sodium channel sensitivity to permethrin and deltamethrin by 1100- and 90-fold, respectively. This may result in the failure of pyrethroid-based dengue vector control.

### Vector control using pyrethroids

In the current study, a combination of bioassays, synergistic testing, and *kdr* genotyping suggested that enhanced metabolizing enzyme (P450) activity, instead of mutations in the sodium channel gene, was involved in the resistance to pyrethroids seen in the *Ae. aegypti* populations collected from Khounkham and Thakhek. In contrast, it was suggested that mutations in the sodium channel gene, rather than enhanced metabolizing enzyme activity, were involved in the resistance to pyrethroids seen in the *Ae. aegypti* population from Xaythany. In other words, it was demonstrated that the mechanism responsible for insecticide resistance to pyrethroids varied among local *Ae. aegypti* populations from different sites in Lao PDR.

In the future, to facilitate efficient and effective vector control using pyrethroids, it will be necessary to elucidate the *kdr* mutations present in the sodium channel genes of local *Ae. aegypti* populations in Lao PDR by *kdr* genotyping and to continuously monitor such populations for the occurrence of certain sodium channel gene mutation haplotypes, especially the P-G-T-F and P-G-T-C haplotypes*.* In addition, it will be necessary to discuss whether it would be appropriate to use synergists, such as PBO, in some areas of Lao PDR. The use of the synergist PBO in combination with deltamethrin could be a useful *Ae. aegypti* management strategy in some areas of Lao PDR.

### Limitations of the present study

In this study, four field populations of *Ae. aegypti* larvae from central Lao PDR were subjected to bioassays, synergist assays, and *kdr* allele genotyping, which provided valuable data about the prevalence and mechanisms of insecticide resistance in these populations. However, this study was affected by the following inherent weaknesses: (1) a comprehensive evaluation of dengue control-related insecticide resistance in *Ae. aegypti* populations requires more quantitative measures of the strength of resistance; i.e., RR_50_ and RR_99_ should be calculated by determining the LC_50_ and LC_99_ ratios of the field and reference strains, and (2) this study involved small sample sizes; i.e., only 3 populations for the bioassays and 4 populations for the *kdr* genotyping; therefore, further investigations involving a larger number of populations are required to confirm the findings of this study.

## Conclusion

The current study revealed the status of insecticide resistance and its underlying mechanisms in *Ae. aegypti* populations from sites in central Lao PDR. Resistance to temephos was observed in the *Ae. aegypti* populations from three sites at the diagnostic dose (0.0286 mg/L) used in the present study. However, this dose represents about one thirty-fifth of the dose recommended by the WHO (1 mg/L) for *Ae. aegypti.* Therefore, it is suggested that, when used according to the WHO guidelines, temephos is still effective for larval control of *Ae. aegypti*. To ensure effective and sustainable vector control is achieved using insecticides in Lao PDR, the rotational use of a mixture of temephos and other insecticides with different mechanisms of action, such as *Bti*, whose effects were confirmed in the current study, and pyriproxyfen, is recommended. In fact, Marombe et al. [17] recommended the use of alternative insecticides with different mechanisms of action when they conducted vector control using insecticides in Lao PDR in 2019, which supports the findings of the present study.

The current study revealed that the mechanisms responsible for resistance to pyrethroids varied among the *Ae. aegypti* populations at the three examined sites. Statistical analyses showed strong associations between the sodium channel gene *kdr* mutations and resistant phenotypes seen in the *Ae. aegypti* population from Xaythany. It was also suggested that increased metabolizing enzyme activity was the main mechanism responsible for the enhanced pyrethroid resistance observed in the Khounkham and Thakhek populations, but not the Xaythany population. A wide variety of metabolizing enzymes, such as P450s, COEs, GSTs, and uridine 5′-diphospho-glucuronosyltransferase, are involved in insecticide metabolism [45–47]. Therefore, the detailed mechanisms responsible for such processes are still insufficiently understood, which is not the case for the processes affected by mutations in the sodium channel gene. In light of this, it will be necessary to analyze metabolizing enzyme genes in multiple populations from Lao PDR using DNA sequencing and/or RNA sequencing analysis involving high-throughput sequencing technologies.

## Supplementary Information

**Additional file 1: Table S1.** Resistance status of *Ae. aegypti* larvae to different insecticides and synergists in Lao PDR. **Table S2.** Comparison of the resistant allele frequency among *Ae. aegypti* larvae from four populations. **Table S3.** Thirteen genotypes and five haplotypes identified in the present study

## Data Availability

The data supporting the conclusions of this article are included within the article and its additional files. The unique DNA haplotype sequences are available from the DNA Data Bank of Japan (DDBJ, accession = LC605641, LC605642, LC605643, LC605644, and LC605645).

## Abbreviations

*Bti*: *Bacillus thuringiensis israelensis*
*kdr*: Knockdown resistance
P450s: Cytochrome P450 monooxygenases
COEs: Carboxylesterases
GSTs: Glutathione S-transferases
Lao PDR: Lao People’s Democratic Republic
DMSO: Dimethyl sulfoxide
WHO: World Health Organization
EA: Ethacrynic acid
PBO: Piperonyl butoxide
TPP: Triphenyl phosphate
PCR: Polymerase chain reaction
ANOVA: Analysis of variance
OR: Odds ratio
95% CI: 95% confidence interval
LC_50_/LC_95_: 50% and 95% lethal concentrations
RR_50_/RR_95_: Resistance ratios obtained at the LC_50_/LC_95_
IGR: Insect growth regulator
