# Supplementary material for: Phenotypic and haplotypic profiles of insecticide resistance in populations of Aedes aegypti larvae (Diptera: Culicidae) from central Lao PDR
Source: Trop Med Health. 2021 Apr 21;49:32. doi: 10.1186/s41182-021-00321-3 (PMC8061177; doi:10.1186/s41182-021-00321-3)
Supplement: Supplementary file 1 — Additional file 1: Table S1. Resistance status of Ae. aegypti larvae to different insecticides and synergists in Lao PDR. Table S2. Comparison of the resistant allele frequency among Ae. aegypti larvae from four populations. Table S3. Thirteen genotypes and five haplotypes identified in the present study [file 41182_2021_321_MOESM1_ESM.docx]

Table S1. Resistance status of *Ae. aegypti* larvae to different insecticides and synergists in Lao PDR

| Larvicides + synergists | Mean percentage mortality±SEM | | | | | |
| --- | --- | --- | --- | --- | --- | --- |
|  | Xaythany | | Khounkham | | Thakhek | |
| Total treated (n) | 272 | | 269 | | 301 | |
| Temephos | 66.7 | ±1.5 | 64.0 | ±7.9 | 89.7 | ±5.6 |
| Temephos + EA | 79.6 | ±6.2 | 63.2 | ±13.2 | 90.2 | ±4.9 |
| Temephos + PBO | 71.5 | ±3.4 | 71.6 | ±7.4 | 72.2 | ±3.3 |
| Temephos + TPP | 85.6 | ±3.4^a^ | 83.0 | ±6.5 | 96.7 | ±10.8 |
| Total treated (n) | 302 | | 283 | | 302 | |
| Deltamethrin | 27.7 | ±8.0^***^ | 5.6 | ±1.2^***^ | 42.7 | ±1.7^****^ |
| Deltamethrin + EA | 20.3 | ±2.9 | 2.8 | ±2.8 | 16.1 | ±3.6 |
| Deltamethrin + PBO | 54.9 | ±11.4 | 100.0 | ±0.0^b^ | 100.0 | ±0.0^b^ |
| Deltamethrin + TPP | 37.1 | ±4.4 | 8.3 | ±2.4 | 23.5 | ±6.1 |
| Total treated (n) | 289 | | 336 | | 356 | |
| Permethrin | 33.4 | ±9.4^***^ | 0.0 | ±0.0^***^ | 7.5 | ±3.2^****^ |
| Permethrin + EA | 7.0 | ±1.5 | 4.1 | ±2.4 | 6.4 | ±4.2 |
| Permethrin + PBO | 52.4 | ±11.3 | 93.0 | ±1.5^b^ | 87.5 | ±1.6^b^ |
| Permethrin + TPP | 10.3 | ±4.9 | 1.4 | ±1.4 | 7.5 | ±4.4 |
| Total treated (n) | 60 | | 64 | | 63 | |
| *Bti* | 100.0 | ±0.0 | 100.0 | ±0.0 | 100.0 | ±0.0 |
| Total treated (n) | 68 | | 73 | | 73 | |
| EA (0.1 mg/L) | 0.0 | ±0.0 | 0.0 | ±0.0 | 0.0 | ±0.0 |
| Total treated (n) | 72 | | 70 | | 69 | |
| TPP (1 mg/L) | 0.0 | ±0.0 | 1.4 | ±1.4 | 0.0 | ±0.0 |
| Total treated (n) | 68 | | 70 | | 71 | |
| PBO (5 mg/L) | 0.0 | ±0.0 | 1.4 | ±1.4 | 0.0 | ±0.0 |

Twenty-five to 30 third- to fourth-instar larvae were used per replication, and 3 to 6 replications were performed for each larvicide and larvicide+synergist combination.

Mean mortality rates followed by an asterisk indicate that the rate for deltamethrin alone or permethrin alone was significantly lower than the mortality rate for temephos alone (***: *p*<0.001, ****: *p*<0.0001, one-way ANOVA followed by Tukey’s multiple comparisons test). Mean mortality rates followed by a superscript letter indicate that the rate for the synergist-treated population was significantly higher than that for the non-synergist-treated population (a: *p*<0.05, b: *p*<0.001, one-way ANOVA followed by Dunnett’s multiple comparisons test).

Table S2. Comparison of the resistant allele frequency among *Ae. aegypti* larvae from four populations

|  | | | | | | | | | | |
| --- | --- | --- | --- | --- | --- | --- | --- | --- | --- | --- |
| 989P allele | | | | |  | 1016G allele | | | | |
| Population |  |  | Significant | Adjusted *p-*value |  | Population |  |  | Significant | Adjusted *p-*value |
| Xaythany | vs. | Pakkading | No | >0.05 |  | Xaythany | vs. | Pakkading | No | >0.05 |
| Xaythany | vs. | Khounkham | Yes | 0.003 |  | Xaythany | vs. | Khounkham | Yes | 0.006 |
| Xaythany | vs. | Thakhek | Yes | <0.0001 |  | Xaythany | vs. | Thakhek | Yes | <0.0001 |
| Pakkading | vs. | Khounkham | Yes | 0.022 |  | Pakkading | vs. | Khounkham | Yes | 0.022 |
| Pakkading | vs. | Thakhek | Yes | <0.0001 |  | Pakkading | vs. | Thakhek | Yes | <0.0001 |
| Khounkham | vs. | Thakhek | Yes | 0.018 |  | Khounkham | vs. | Thakhek | Yes | 0.018 |
| 1520I allele | | | | |  | 1534C allele | | | | |
| Population |  |  | Significant | Adjusted *p-*value |  | Population |  |  | Significant | Adjusted *p-*value |
| Xaythany | vs. | Pakkading | No | >0.05 |  | Xaythany | vs. | Pakkading | No | >0.05 |
| Xaythany | vs. | Khounkham | No | >0.05 |  | Xaythany | vs. | Khounkham | Yes | <0.0001 |
| Xaythany | vs. | Thakhek | No | >0.05 |  | Xaythany | vs. | Thakhek | Yes | <0.0001 |
| Pakkading | vs. | Khounkham | No | >0.05 |  | Pakkading | vs. | Khounkham | Yes | 0.0031 |
| Pakkading | vs. | Thakhek | No | >0.05 |  | Pakkading | vs. | Thakhek | Yes | 0.0004 |
| Khounkham | vs. | Thakhek | No | >0.05 |  | Khounkham | vs. | Thakhek | No | >0.05 |

*P*-values were calculated using Fisher’s exact test with Holm’s correction for multiple comparisons.

Table S3. Thirteen genotypes and five haplotypes identified in the present study

| Genotype | Haplotype |
| --- | --- |
| S989P-V1016G-T150I-F1534C | S989P-V1016G-T150I-F1534C |
| S/S+V/V+T/T+F/F | S-V-T-F |
| S/S+V/V+T/T+F/C | S-V-T-C |
| S/S+V/V+T/T+C/C |  |
| S/S+V/V+T/I+F/C | S-V-I-C |
| S/S+V/V+T/I+C/C |  |
| S/S+V/V+I/I+C/C |  |
| P/P+V/G+T/T+F/F | P-G-T-F |
| P/P+G/G+T/T+F/F |  |
| S/P+V/G+T/T+F/C | P-G-T-C |
| S/P+G/G+T/T+F/C |  |
| P/P+V/G+T/T+C/C |  |
| P/P+G/G+T/T+F/C |  |
| P/P+G/G+T/T+C/C |  |

The sites of the *kdr* mutations are underlined.
